# Radiation therapy attenuates lymphatic vessel repair by reducing VEGFR-3 signalling

**DOI:** 10.3389/fphar.2023.1152314

**Published:** 2023-04-28

**Authors:** Vinochani Pillay, Lipi Shukla, Prad Herle, Simon Maciburko, Nadeeka Bandara, Isabella Reid, Steven Morgan, Yinan Yuan, Jennii Luu, Karla J. Cowley, Susanne Ramm, Kaylene J. Simpson, Marc G. Achen, Steven A. Stacker, Ramin Shayan, Tara Karnezis

**Affiliations:** ^1^ O’Brien Institute Department, St Vincent’s Institute for Medical Research, Fitzroy, VIC, Australia; ^2^ Department of Plastic Surgery, St. Vincent’s Hospital, Fitzroy, VIC, Australia; ^3^ Faculty of Health Sciences, ACU, AORTEC; Australian Catholic University, Fitzroy, VIC, Australia; ^4^ Victorian Centre for Functional Genomics, Peter MacCallum Cancer Centre, Melbourne, VIC, Australia; ^5^ Department of Biochemistry and Pharmacology, University of Melbourne, Parkville, VIC, Australia; ^6^ Department of Medicine, University of Melbourne, St. Vincent’s Hospital, Fitzroy, VIC, Australia; ^7^ Tumour Angiogenesis and Microenvironment Program, Peter MacCallum Cancer Centre, Melbourne, VIC, Australia; ^8^ Department of Surgery, Royal Melbourne Hospital, The University of Melbourne, Parkville, VIC, Australia; ^9^ Department of Plastic Surgery, Alfred Health, Melbourne, VIC, Australia

**Keywords:** radiotherapy, VEGFR-3, VEGF-C, lymphoedema, radiation-injury, LEC, lymphatics

## Abstract

**Introduction:** Surgery and radiotherapy are key cancer treatments and the leading causes of damage to the lymphatics, a vascular network critical to fluid homeostasis and immunity. The clinical manifestation of this damage constitutes a devastating side-effect of cancer treatment, known as lymphoedema. Lymphoedema is a chronic condition evolving from the accumulation of interstitial fluid due to impaired drainage via the lymphatics and is recognised to contribute significant morbidity to patients who survive their cancer. Nevertheless, the molecular mechanisms underlying the damage inflicted on lymphatic vessels, and particularly the lymphatic endothelial cells (LEC) that constitute them, by these treatment modalities, remain poorly understood.

**Methods:** We used a combination of cell based assays, biochemistry and animal models of lymphatic injury to examine the molecular mechanisms behind LEC injury and the subsequent effects on lymphatic vessels, particularly the role of the VEGF-C/VEGF-D/VEGFR-3 lymphangiogenic signalling pathway, in lymphatic injury underpinning the development of lymphoedema.

**Results:** We demonstrate that radiotherapy selectively impairs key LEC functions needed for new lymphatic vessel growth (lymphangiogenesis). This effect is mediated by attenuation of VEGFR-3 signalling and downstream signalling cascades. VEGFR-3 protein levels were downregulated in LEC that were exposed to radiation, and LEC were therefore selectively less responsive to VEGF-C and VEGF-D. These findings were validated in our animal models of radiation and surgical injury.

**Discussion:** Our data provide mechanistic insight into injury sustained by LEC and lymphatics during surgical and radiotherapy cancer treatments and underscore the need for alternative non-VEGF-C/VEGFR-3-based therapies to treat lymphoedema.

## 1 Introduction

A combination of early detection, surveillance and refined cancer therapies has led to improved overall cancer survival rates (AIHW, 2019). This improvement has, however, provided greater opportunity for the unwanted effects of cancer treatments, including the progressive, often late-onset soft-tissue injury caused by radiotherapy (Dormand et al., 2005), which can lead to lymphoedema. Lymphoedema is caused by impaired function of the lymphatics (Szuba and Rockson, 1998; Szuba et al., 2002), a hierarchical network of vessels lined with LEC, which commence as thin-walled initial lymphatics in the superficial dermis that drain into deeper collecting lymphatics (Shayan, 2006) and then into lymph nodes. Over half of patients diagnosed with solid tumours require radiotherapy as a primary or adjunctive cancer treatment alongside surgery (Barton et al., 2014). It is well known that radiotherapy is an independent risk factor for lymphoedema (Allam et al., 2020), however the risk is amplified when combined with surgical lymph node clearance, resulting in a lymphoedema incidence of up to 40% in the upper limb and 60% in the lower limb (Starritt et al., 2004; Armer and Stewart, 2010; Ugur et al., 2013; Warren et al., 2014; Allam et al., 2020; Kim et al., 2021). The exact reason behind this additive effect is not well understood.

Radiotherapy works by exerting a lethal anti-tumour effect on rapidly dividing tumour cells (Ryan, 2012). The pathological effects of radiation injury were previously attributed to obliterated blood vessels, however, more recently, the pathophysiology of radiation exposure has been shown to be more complicated (Tibbs, 1997). Radiotherapy impairs lymphatic function (Avraham et al., 2010; Singh et al., 2023) and over time, lymphoedema also results in progressive scarring, reduced immunity, and recurrent local and systemic infection—the incidence of cellulitis in lymphoedema patients is up to 50%, cf. a rate of 2%–5% in the normal population (Aldrich et al., 2020). The fibrosis and tissue impairment resulting from lymphoedema creates a “vicious cycle” that further compromises lymphatic function, and irrespective of the cause, may independently lead to impaired healing (Tibbs, 1997; Bhide et al., 2012; Momoh et al., 2013) that may expose vital structures or underlying implants or necessitate more complex reconstructive surgery, with poorer outcomes (Herle et al., 2015). There is currently no “cure” for lymphoedema, and treatment options consist of massage therapy, physical compression and—for a select group of patients—surgical options may be considered such as lymph node transfer (Tang et al., 2021), lymphaticovenous anastomosis and liposuction (Leppäpuska et al., 2022). Therefore, novel biological treatments for lymphoedema are critically needed. Whilst anecdotal clinical reports of treating lymphoedema using imported undamaged tissue (fat grafting or free vascularised tissue) demonstrate the potential benefits of alleviating lymphoedema (Tang et al., 2021), the exact mechanisms of such putative effects remain unknown.

During injury, specifically surgical insults, lymphatic regeneration occurs either by sprouting from, or remodelling of, pre-existing lymphatic vessels via a process known as lymphangiogenesis. Lymphangiogenesis is regulated by members of the Vascular Endothelial Growth Factor (VEGF) family, namely, VEGF-C and VEGF-D (Joukov et al., 1996; Achen et al., 1998; Haiko et al., 2008), and involves proliferation, sprouting, migration and tube formation by Lymphatic Endothelial Cells (LEC), processes that are driven by these growth factors via signalling downstream of their cognate tyrosine kinase receptor, VEGFR-3 (Srinivasan and Oliver, 2011; Bowles et al., 2014). Targeting blood or lymphatic vessels using a variety of therapeutic strategies has been tested as an approach to treat numerous disease conditions, such as cancer, ischemic disorders, and tissue oedema (Norrmén et al., 2011; Stacker et al., 2014). Several groups have investigated the utility of lymphangiogenic growth factors such as VEGF-C in overcoming the effects of radiotherapy injury to LEC, aiming to mitigate the clinical outcome of lymphoedema (Goldman et al., 2005; Padera et al., 2008; Kesler et al., 2013; Padera et al., 2015). Taken together, these studies suggest that lymphangiogenic growth factors are unable to exert a protective or sustained functional lymphangiogenic effect to counteract the effects of radiotherapy; however, a mechanistic explanation for this effect is unclear.

Here, we investigated molecular mechanisms underlying the deleterious effects of radiotherapy on lymphatic function *in vivo* and *in vitro*. We interrogated LEC functions in cell-based assays that replicate cellular processes required for lymphangiogenesis and analysed the effects of lymphangiogenic growth factors on irradiated LEC, both *in vitro* and in animal models of lymphatic injury, allowing us to better understand the influences elicited by radiation on the VEGF-C/VEGF-D/VEGFR-3 axis. Importantly, our findings provide mechanistic insight into clinical limitations of using VEGF family lymphangiogenic growth factors for lymphoedema therapy.

## 2 Materials and methods

### 2.1 Ethics/tissue collection

Human tissue samples were collected from patients undergoing delayed reconstruction for cancer treatment at St. Vincent’s Hospital Melbourne or St. Vincent’s Private Hospital (Fitzroy and East Melbourne, Australia). Patients were consented prior to tissue collection in accordance with ethics protocol HREC No. 52/03. 6–8-week-old mice were used for all experiments with ethical approval from the Animal Ethics Committee at St Vincent’s Hospital Melbourne (AEC 015/5/r5 and AEC 016/016/r1).

### 2.2 Cell culture

Human adult dermal lymphatic endothelial cells (LEC) were from Promocell (Germany) (CC-12217), and complete Endothelial Cell Growth Medium MV2 containing 5% FCS plus growth factors and supplements (prepared by combining Basal Media with Supplement Pack supplied in the kit) was from Promocell (Germany) (CC-22121). Cells were grown in tissue culture dishes and plates coated with human fibronectin [5 μg/mL, Sigma-Aldrich (United States) #F2006] and used at passage numbers 4–7. Complete, growth factor-free and serum-reduced growth factor-free media solutions were endothelial basal medium MV2 [PromoCell (Germany)] with the addition of 5% fetal calf serum (FCS) and supplement pack, 5% FCS only and 2% FCS only [PromoCell (Germany)], respectively. Cells were incubated at 37°C in a humidified atmosphere of 5% CO_2._

### 2.3 Cell irradiation and dosing regimes

Cells were irradiated at the Bio-resources Centre (Victoria, Australia) using a Gammacell^®^ 40 Irradiator (Best Theratronics, Canada). This machine delivered 0.9967584 Gy/min with the chosen gamma irradiation dose evenly delivered across the irradiation drawer. Dose Uniformity (typical) was ±7% over a 260 mm diameter and 100 mm height chamber. Cells were irradiated using a single dose of 10 Gy, a dose that is reflective of clinical irradiation of cells in the dermal layer of skin (Possenti et al., 2021), whereas control cells received 0 Gy (no radiotherapy).

### 2.4 Proliferation assay

Proliferation assays were performed at the Victorian Centre for Functional Genomics, Peter MacCallum Cancer Centre, Victoria, Australia. To test the effect of radiotherapy on LECs, cells were seeded at a density of 2,000 cells/well in fibronectin-coated black-walled 96-well tissue culture plates in 100 µL of Complete Media and allowed to attach overnight. Cells received either 0 Gy (control) or 10 Gy (treatment) doses of ionising radiation. Media was replaced with fresh serum-reduced growth factor-free media after treatment. Cells were allowed to proliferate for 72 h before fixation (4% PFA) and stained with DAPI for automated imaging. Cell nuclei were counted via high throughput imaging (Cellomics VTI Arrayscan, Thermo Fisher Scientific, United States). To test the effect of radiotherapy on responsiveness of LECs to lymphangiogenic growth factors (VEGF-C and VEGF-D), the same method as described above was used and after 0 Gy or 10 Gy treatment, media was removed and replaced with serum-reduced growth factor-free media containing VEGF-C (Opthea, Australia) or VEGF-D (Opthea, Australia). Cells were allowed to proliferate for 72 h and then fixed, stained, imaged, and counted.

### 2.5 Scratch migration/wound healing assay

Scratch migration assays were performed at the Victorian Centre for Functional Genomics, Peter MacCallum Cancer Centre, Victoria, Australia. Cells were plated at a density of 75,000 cells/well (to achieve about 90% confluence) in fibronectin-coated black-walled 96-well tissue culture plates in 100 µL of complete media and allowed to attach overnight. Duplicate plates for each treatment were prepared for T0 and T24 endpoints. Cells received either 0 Gy or 10 Gy doses of irradiation. Media was replaced with complete media and cells were incubated for a further 48 h. A 96-pin wounding device with “FP” pins (V&P Scientific Inc., United States) controlled by a workstation robot (Sciclone ALH 3000, Caliper Life Sciences, Thermo Fisher Scientific, United States) was used to create uniform scratches (approximately 3.8 mm long × 0.38 mm wide) in the cell monolayer. Cells were gently washed and medium replaced. Growth factors and controls were added to the T24 plate. Cells were fixed and T0 and T24 plates stained with DAPI, Rhodamine Phalloidin and CellMask Green and then imaged (Cellomics VTI Arrayscan, Thermo Fisher Scientific, United States). The percentage gap closure was calculated as follows: % Gap closure = Area migrated at T24/Area at T0 × 100%.

### 2.6 Tube formation

Forty-eight-well tissue culture plates were pre-coated with a thick layer of Growth Factor Reduced Matrigel (according to manufacturer protocols, BD Biosciences, United States) after which 0 Gy and 10 Gy-treated LEC (48 h post treatment) were seeded in complete media. At 48 h using bright field microscopy at x10 objective on the Olympus IX71 Inverted Microscope (Olympus, United States), photographs were taken and quantified using the Lymphatic Vessel Analysis Protocol (LVAP) plug-in (Shayan et al., 2007) for ImageJ (FIJI open-source software, United States) using parameters of tubes and branches.

### 2.7 Chemotaxis/boyden chamber assay

Chemotaxis assays were performed in 96-well plates with 8.0 µm Pore High density PET membrane (FluoroBlok 96-well system, BD Falcon, United States). The basal chamber was filled with 150 μL of growth factor free media for baseline 0 Gy vs. 10 Gy experiments. Serum reduced growth factor free media was used as the media for growth factor experiments with 200 ng/mL VEGF-C or VEGF-D (Opthea, Australia). 0 Gy- and 10 Gy-treated LEC (48 h post treatment) were seeded at 5,000 cells/well in 75 μL of serum starved media in the apical membrane (to allow for creation of a chemotactic gradient) and incubated for 24 h. Media in the basal chamber was replaced with PBS to wash the PET membrane basal surface, where transmigrated cells were attached. The basal surface of the membrane was fixed with 4% PFA, washed with PBS, stained with DAPI, and stored in PBS (4°C). The entire basal surface of the membrane was imaged [BX53 Semi-Motorised Olympus fluorescent Microscope (Olympus, United States)] at x4 objective. ImageJ was used to quantify the number of DAPI stained nuclei by automated single colour image counting. Briefly, the image was converted from RGB to 16-bit greyscale, image threshold was selected and adjusted to highlight all DAPI stained nuclei and exclude any additional fluorescence. The image was then converted to a binary image and particles analysed. This method therefore quantified the number of cells transmigrated from the apical to the basal side of the membrane and was represented as mean fluorescence intensity.

### 2.8 Sprouting assay

Dry Cytodex-3 Beads (Cytiva, United Kingdom) were hydrated in PBS (Sigma-Aldrich, United States) and resuspended (30,000 beads/mL) and stored in 4°C as per manufacturer’s instructions. A 10 cm plate of cultured LECs at 80% confluence was trypsinised and 2,500 prepared Cytodex-3 Beads washed and resuspended with 1 × 10^6^ of the LEC in a FACS tube for 4 h. Coated beads were transferred to a T25 flask in 5 mL of complete media and left overnight. Two mg/mL fibrinogen type 1 (Sigma-Aldrich, United States) was added to 0.15 units/mL aprotinin (Sigma-Aldrich, United States). LEC-coated beads were resuspended in complete media then washed before being counted on a coverslip and resuspended in the fibrinogen/aprotinin solution (concentration 500 beads/mL). 0.625 units/mL thrombin (Sigma-Aldrich, United States) and 0.5 mL of the fibrinogen/bead solution were added to 10 wells of a 24-well plate then, after settling for 5 min (RT), were transferred to a humidified incubator for 15 min. If applicable, the plate was then treated with ionising radiation (10 Gy). Normal human dermal fibroblasts (PromoCell, Germany) were seeded onto each well at a concentration of 20,000 cells/well before plates were returned to a humidified incubator. Culture medium was changed 48 hourly and the experiment terminated at day 12 by fixation (4% PFA) and then staining with 1:1000 DAPI and 1:500 phalloidin (30 min) at RT. Plates were imaged [Nikon A1R confocal microscope (Nikon, Japan)] at x10 objective and images processed and quantified using the FIJI open-source software (FIJI open-source software, United States).

### 2.9 Western blotting

For the detection of total and phosphorylated VEGFR-2 and VEGFR-3 and downstream signalling molecules, cells were seeded at a density of 5,000 cells/cm^2^ in either 10 cm or 6 cm fibronectin-coated tissue culture dishes and grown to approximately 90% confluence. Cells received either a 0 Gy (control) or 10 Gy (treatment) dose of ionising radiation. Media was removed and replaced with fresh complete media and cells incubated for a further 48 h. Cells were washed with serum-free media and serum starved for about 4 h prior to stimulation with either VEGF-A (50 ng/mL for 5 min), VEGF-C (200 ng/mL for 10 min) or VEGF-D (200 ng/mL for 10 min). Cells were washed twice in ice-cold PBS and lysed with RIPA buffer (Sigma-Aldrich, United States) containing protease and phosphatase inhibitors. Lysates were collected by scraping and frozen at −80°C. Thawed lysates were incubated with rotation at 4°C for 10–15 min and clarified by centrifugation at 4°C before protein was quantified using the BCA Rapid Gold Kit (Thermo Fisher Scientific, United States). Total protein (15–20 µg) was separated on NuPAGE 3%–8% Tris-Acetate gels (Thermo Fisher Scientific, United States) and transferred to PVDF membranes at 25 V for 10 min using the iBlot2 Dry Blotting System (Thermo Fisher Scientific, United States). Blocking steps and antibody incubations were performed in Odyssey Blocking Buffer—TBS or Intercept Blocking Buffer - TBS (LI-COR Biosciences, United States) and blots washed in TBS-Tween containing 0.1% Tween-20. Primary antibodies for detection of VEGFR-2 (clone 55B11, # 2479S), phospho-VEGFR-2 (Tyr1175, clone 19A10, #2478), AKT (pan, clone C67E7, #4691S), phospo-AKT (S473, clone D9E, YP, R, # 4060S), P44/42 MAPK (Erk1/2) (clone 137FS, # 4695P) and phospho-p44/42 MAPK (Erk1/2) (T202/Y284, #9101S) were purchased from Cell Signalling Technology. Primary antibodies for the detection of VEGFR-3 (clone 9D9F9, mAb 3757) and phospho-VEGFR-3 (Tyr1230/1231, clone CY115) were obtained from Merck (United States) and Cell Applications (United States), respectively. Primary antibodies for detection of β-actin (clone C4, #SC-47778) and neuropilin-2 (clone C-9, #SC-13117) were purchased from Santa Cruz Biotechnology (United States). Secondary antibodies were either IRDye 800CW goat anti-mouse or IRDye 680RD goat anti-rabbit IgG (LI-COR Biosciences, United States). Western blots were imaged using the Odyssey CLx Imaging System (LI-COR Biosciences, United States). The pR3/tR3 ratio was calculated from quantification of the optical densities of the signals from phosphorylated and total VEGFR3, normalised to the signals from actin loading control, using Image Studio Software supplied with Odyssey CLx Imaging System (LI-COR Biosciences, United States).

### 2.10 Gene expression analysis by quantitative PCR

To determine gene expression levels for VEGFR-3 (FLT4) by quantitative PCR (qPCR), LECs were grown on fibronectin coated 12-well cell culture plates at a density of 6,500 cells/cm^2^ in complete medium. Once cells reached approximately 95% confluency, cells received either a 0 Gy (control) or 10 Gy (treatment) radiation. At 48 h after irradiation, total RNA was extracted using RNeasy mini kit (Qiagen, Germany) per the manufacturer’s instructions and the concentration of RNA was determined using a Nanodrop Spectrophotometer (Thermo Fisher Scientific, United States). cDNA was synthesized from 500 ng RNA using a High-Capacity Reverse Transcription Kit (Thermo Fisher Scientific, United States). VEGFR-3 gene expression was determined by qPCR performed with a Quant Studio™ 6 Flex Real-Time PCR System (Thermo Fisher Scientific, United States) using a human VEGFR-3 specific TaqMan prob (Hs01047677; Thermo Fisher Scientific, United States) and TaqMan gene expression master mix (Thermo Fisher Scientific, United States). Target gene expression was normalized to human GAPDH mRNA levels (human GAPDH specific TaqMan prob; Hs02786624; Thermo Fisher Scientific, United States) using the ΔΔCt method using the software integrated into the real time thermal cycler.

### 2.11 *In vivo* ear model experiment

The effects of radiotherapy on lymphangiogenesis in wound healing were determined using an *in vivo* ear wound model in Prox-1/GFP mice (Choi et al., 2012). The mice were anaesthetised with intraperitoneal injection of Water +10% Ketamine [100 mg/kg] + 10% Xylazine [10 mg/kg], at a dose of 0.1 mL/10 mg body weight. Briefly, mice under anaesthetic sedation were treated with 10 Gy irradiation of their right ears while their shielded left ear served as a control (0 Gy). A surgical wound was created 1 week after irradiation using a 2 mm punch biopsy and mice were culled at the 1, 2 or 3-week post-wounding timepoints. For VEGF treatment, growth factors were injected locally in the ear thrice in the first week following punch biopsy. The ear tissue was dissected, fixed and whole-mounted for imaging with confocal microscopy (Nikon A1R confocal microscope). The GFP expressing lymphatic vessels were quantified using ImageJ and a macro (Arganda-Carreras et al., 2010) and ear lymphatic sprouting was analysed using the LVAP (Shayan et al., 2007).

### 2.12 *In vivo* tail model experiment

The effects of radiotherapy on lymphangiogenesis in wound healing were determined using utilising an *in vivo* tail model in Prox-1/GFP mice. The tail model allowed interrogation of lymphatic architecture and function distal to the zone of injury. Radiotherapy was conducted under anaesthetic, with animal tails being placed in the centre of a lead-perspex jig and a central lead shield used to shield all but the proximal 20 mm of the mouse tail, thus achieving selective radiation of the proximal tail. One week following 10 Gy radiotherapy (or 0 Gy control radiotherapy) surgery was performed in selected groups. Surgery involved removing a 10 mm long patch of skin and subcutaneous tissue from the proximal tail, starting at a point 10 mm from the base of the tail. Following this, patent blue stained collecting lymphatic vessels (CLVs) were dissected away from the major blood vessels of the tail and disrupted with bipolar cautery and microsurgical scissors. Animals were culled 4 weeks after intervention and tissues harvested from the wound zone and from the distal tail, and subsequently fixed and whole-mounted for imaging with confocal microscopy, as above.

### 2.13 Bioassays for binding and cross-linking of extracellular domains of VEGFR-2 or VEGFR-3

To determine growth factor potency, bioassays based on cell lines expressing chimeric receptors consisting of the entire extracellular domain of mouse VEGFR-2 or human VEGFR-3 and the trans-membrane and cytoplasmic domains of the mouse erythropoietin receptor were used (Stacker et al., 1999). Binding and cross-linking of the chimeric receptors allows these cells to survive and proliferate in the absence of IL-3. DNA synthesis or proliferation of cells was monitored using a ViaLight Plus kit (Lonza, Basel, Switzerland), or Presto Blue™ cell viability reagent (Invitrogen) according to the manufacturers’ protocols.

### 2.14 Human Phospho-RTK arrays

Cells were seeded in complete medium in 10 cm fibronectin-coated tissue cultures dishes at a density of 5,000 cells/cm^2^ and allowed to reach confluence. Cells received either a 0 Gy (control) or 10 Gy (treatment) dose of radiation. Media was replaced with fresh complete media and cells incubated for a further 48 h. Cells were washed with serum-free medium and serum starved for 4 h prior to stimulation with a growth factor mixture consisting of VEGF-A (50 ng/mL for 5 min), VEGF-C (200 ng/mL for 10 min) or VEGF-D (200 ng/mL for 10 min), EGF [100 ng/mL for 5 min, PromoCell (Germany)], bFGF [100 ng/mL for 5 min, (PromoCell, Germany)], IGF-1 [100 nM for 5 min, (PromoCell, Germany)] and heparin (1 μg/mL for 5 min; added 5 min before the addition of bFGF). Cells were lysed and analysed with the human Phospho RTK-array kit (R&D Systems, United States) as per manufacturer’s instructions.

### 2.15 Statistical analysis

Statistical analysis of differences between experimental groups and controls were conducted utilising a Student’s *t*-test or one-way ANOVA, with or without multiple group comparisons, where indicated. A *p*-value <0.05 was considered statistically significant (GraphPad Prism 6.0, United States).

## 3 Results

### 3.1 Radiation reduces lymphangiogenesis and impairs lymphatic function after surgical wounding *in vivo*

We sought to uncover the mechanisms driving the clinical findings of significant effects of radiotherapy on tissues and the associated incidence of lymphoedema. To address this question, we established mouse models of injury to lymphatic vessels that mimic the injuries inflicted during cancer treatments (surgery and radiotherapy). A dose of 10 Gy single beam radiotherapy was used to mimic the clinical circumstances in which normal tissue exposure to multi-beam beam radiation is estimated to be up to 60% of a 50 Gy –70 Gy dose range (Kry et al., 2012; Stewart et al., 2012; Possenti et al., 2021). This is also represented in Figure 1 of Shukla et al’s diagram of normal tissue radiotherapy injury (Shukla, 2015). Our models employed well-established murine lymphoedema models in Prox-1/GFP mice (Choi et al., 2012), due to the ease of imaging and quantifying lymphatics in thin mouse dermis and the green lymphatics vessels visualised using blue fluorescent light.

**FIGURE 1:**
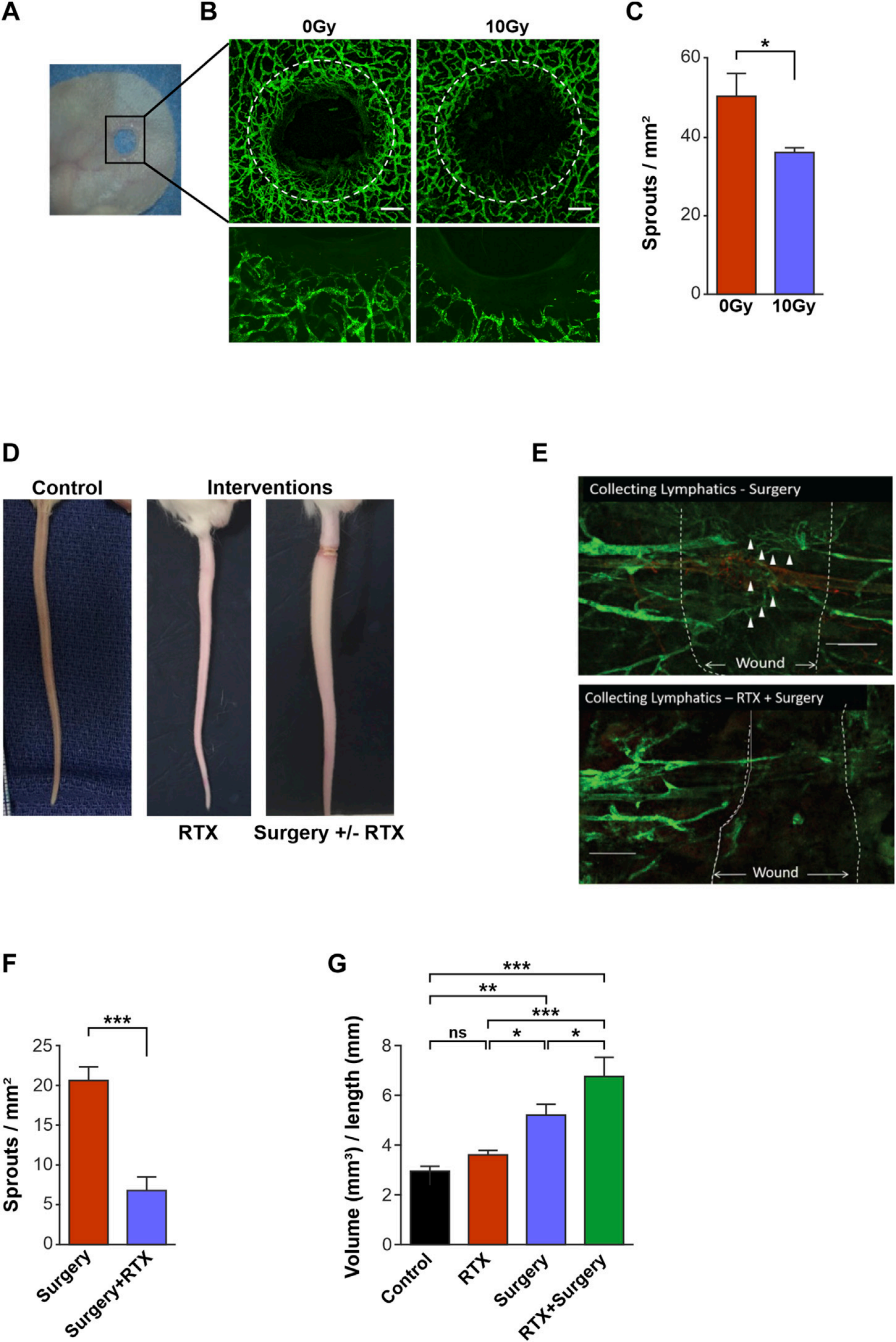
Radiotherapy reduces lymphangiogenesis and impairs lymphatic function *in vivo.* **(A)** Photograph showing the ear wound model in the PROX1-GFP1 mouse. **(B)** Confocal images of 0 Gy Control and 10 Gy irradiated ears 3 weeks post-treatment in the PROX1-GFP mouse. Photos with x4 objective (top images) and high objective images with x20 objective (bottom). Lymphatics are green, white dashed lines indicate wound edges and scale bars denote 200 μm. **(C)** Quantification of lymphatic sprouts on the regenerating wound edge in 0 Gy and 10 Gy ears at the 3-week timepoint. **(D)** Photographs showing a normal (non-operated, non-irradiated) tail (left) and a lymphoedematous tail resulting from interventions of radiotherapy (RTX) and surgery ± RTX. **(E)** Confocal microscopy, x10 objective, of the surgical tail model demonstrating lymphangiogenesis (∆) across the surgical wound in the surgery only setting (top) and the radiotherapy (RTX) with surgery setting (bottom), with white dashed lines denoting the edges of the surgical wounds. **(F)** Quantification of lymphatic sprouts across the surgical wound comparing surgery alone vs. RTX with surgery. **(G)** Tail volume quantification to demonstrate differing degrees of lymphoedema after interventions of control, RTX alone, surgery alone and RTX with surgery. All assays performed a minimum of 3 times (with at least 3 technical replicates per assay). Data expressed as mean, error bars represent SEM. *p*-values calculated using Student’s *t*-test (panels C and F) or one way ANOVA (panel G) (*<0.05; **p < 0.01; ***p < 0.0001, ns = not significant).

Mouse ears were irradiated both in their native and wounded states (Figure 1A). We detected no difference in lymphatic vessel density between irradiated or control ears in the *unwounded* groups (Supplementary Figures S1A, B), which was corroborated by findings in analogous human tissue (Supplementary Figures S1C, D). However, in the model of ear wounding, avid lymphangiogenesis was seen to arise from pre-existing lymphatics surrounding unirradiated mouse ear wounds (Figures 1A, B left panel). In contrast, when wounded ears were also irradiated with 10 Gy (Figure 1B right panel), lymphangiogenesis was significantly restricted compared to wounded but unirradiated 0 Gy control ears, at a 3-week timepoint (Figure 1C).

To mimic the clinical situation, we used a mouse tail lymphedema model to interrogate the effects of radiotherapy on the lymphatic vessels in surgical wounding (Figure 1D). Tail wound analysis showed lymphatic vessels bridging the wound gap in non-irradiated wounded animals (Figure 1E); whilst both the numbers of vessel sprouts and vessels that traversed the wound were markedly reduced in tails that were also subjected to radiation (Figures 1E, F).

Further, the volumes of the tails that were treated with surgery, radiotherapy, or a combination of the two interventions were measured as an indicator of the development of lymphoedema in each setting (Figure 1G). Importantly, radiotherapy alone did not lead to significant differences in tail volumes compared to controls. In contrast, surgery wounding resulted in a significant increase in tail volume, compared to unwounded control tails (mean tail volumes of 5.26 mm^3^ cf. 2.99 mm^3^ in controls, *p* < 0.01) (Figure 1G). Meanwhile, surgery in combination with radiotherapy demonstrated an even greater increase in tail volume compared to control tails (mean tail volumes 6.82 mm^3^ cf. 2.99 mm^3^ in controls, *p* < 0.01), demonstrating the additive effect of both injury modalities (Figure 1G).

Taken together, these *in vivo* models demonstrated that whilst radiotherapy alone does not demonstrate marked alteration to intact lymphatic vessels, the addition of radiation to lymphatic vessels that are wounded restricts lymphangiogenesis in both the ear and the tail murine models. Critically, this restriction of lymphangiogenesis may result in a significant increase in mouse tail volume in the tail lymphoedema model.

### 3.2 Radiotherapy selectively affects key lymphatic endothelial cell functions *in vitro*

Having shown that radiotherapy restricted lymphangiogenesis in the mouse ear and tail wounding models, we next sought to investigate whether the individual cell functions that make up lymphangiogenesis (Shayan et al., 2007; Alitalo, 2011; Oliver et al., 2020) were specifically impaired when exposed to radiotherapy. *In-vitro* functional assays were performed to investigate LEC proliferation, migration, sprouting and tube formation in response to radiotherapy injury.

**FIGURE 2:**
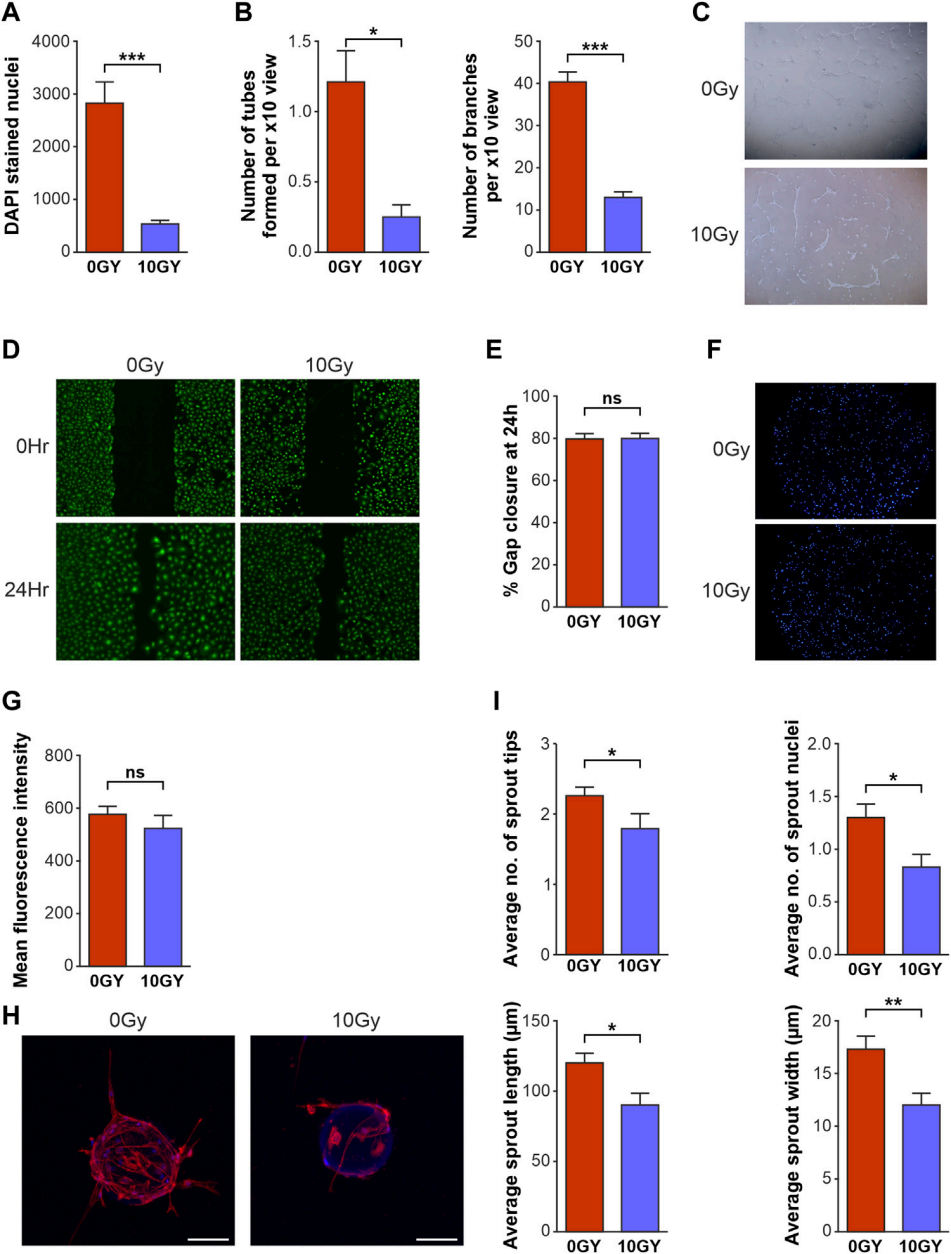
Radiotherapy differentially affects key functional activities of LEC *in vitro*. Quantification of the effects of 10 Gy radiotherapy on LEC **(A)** proliferation after 48 h, **(B)** tube formation as assessed by number of tubes and branches per x10 objective view at 48 h. **(C)** Bright field imaging of LEC seeded on GFR Matrigel photographed at 48 h after radiotherapy. 0 Gy control LEC at 48 h x10 objective demonstrating organised and well-formed tubes while 10 Gy irradiated cells display reduced tube formation with disorganised structure. **(D)** Fluorescence imaging of 2D scratch wound migration assays with representative images of 0Gy and 10 Gy LEC at 0 h and 48 h. **(E)** Graphical representation of 2D scratch wound migration % gap closure at 24 h. **(F)** DAPI stained nuclei fluoresence imaging representing chemotaxis of LEC at 24 h. **(G)** Graphical representation of chemotaxis at 24 h. **(H)** Confocal imaging of LEC spheroids photographed 12 days post seeding with x10 objective, where the 0 Gy Non-irradiated spheroid (left) shows an increased number of sprouts with multiple nuclei and increased length and width compared to irradiated (10 Gy) spheroid (right). **(I)** Spheroid sprouting parameters quantified at 12 days. All assays performed a minimum of 3 times (with at least 3 technical replicates per assay). Asterisks above bar graphs indicate statistical significance. Data expressed as mean with error bars representing SEM. *p* values were calculated using Student’s *t*‐test. (*p < 0.05; **p < 0.01; ***p < 0.0001, ns = not significant).

LEC proliferation assays involved quantification of DAPI-stained nuclei of the LEC within wells that were either irradiated or treated with 0 Gy radiation, under identical conditions. These assays demonstrated that 10 Gy radiotherapy significantly reduced proliferation of LEC, compared with the number of LEC seen in the 0 Gy control wells (Figure 2A). In tube formation assays, control (0 Gy) LEC formed many branches per x10 field (Figures 2B, C), whilst irradiated (10 Gy) LEC formed significantly fewer branches. Quantification of the number of tubes formed in the same assay demonstrated a similar reduction in tube number, following exposure to radiotherapy (Figures 2B, C), with parameters quantified in accordance with the LVAP protocol (Shayan et al., 2007). To assess LEC migration, a 2D scratch wound model of cell migration in a monolayer (Figure 2D) was utilised. The results of this experiment showed no significant difference between the migration of the 0 Gy control LEC and the 10 Gy irradiated LEC, at 24 h following the scratch intervention (Figure 2E). Similarly, a LEC chemotaxis cell migration model showed no difference between the control unirradiated (0 Gy) (Figure 2F top panel) and irradiated (10 Gy) (Figure 2F bottom panel) LEC groups, in terms of their migration response along a growth factor gradient (Figure 2G).

Finally, a 3D spheroid-sprouting assay was performed to study *in vitro* sprouting characteristics (sprout tip number, nuclei, length, and width) after radiotherapy (Figure 2H). Critically, quantification by confocal microscopy showed that radiation treatment resulted in reduction of all parameters of LEC sprouting, namely, the number of sprout tips; number of sprout nuclei; average sprout length; and width of sprouts (Figure 2I).

Taken together, our platform of functional assays demonstrated a *selective* diminution of key LEC lymphangiogenic functions. Sprouting, branching, tube formation and proliferation - key parameters that constitute the processes of functional lymphangiogenesis - were significantly restricted by radiotherapy treatment. Interestingly, LEC migration and chemotaxis were not impaired by radiotherapy in our assays.

### 3.3 Radiotherapy selectively impairs LEC responsiveness to lymphangiogenic growth factors

**FIGURE 3:**
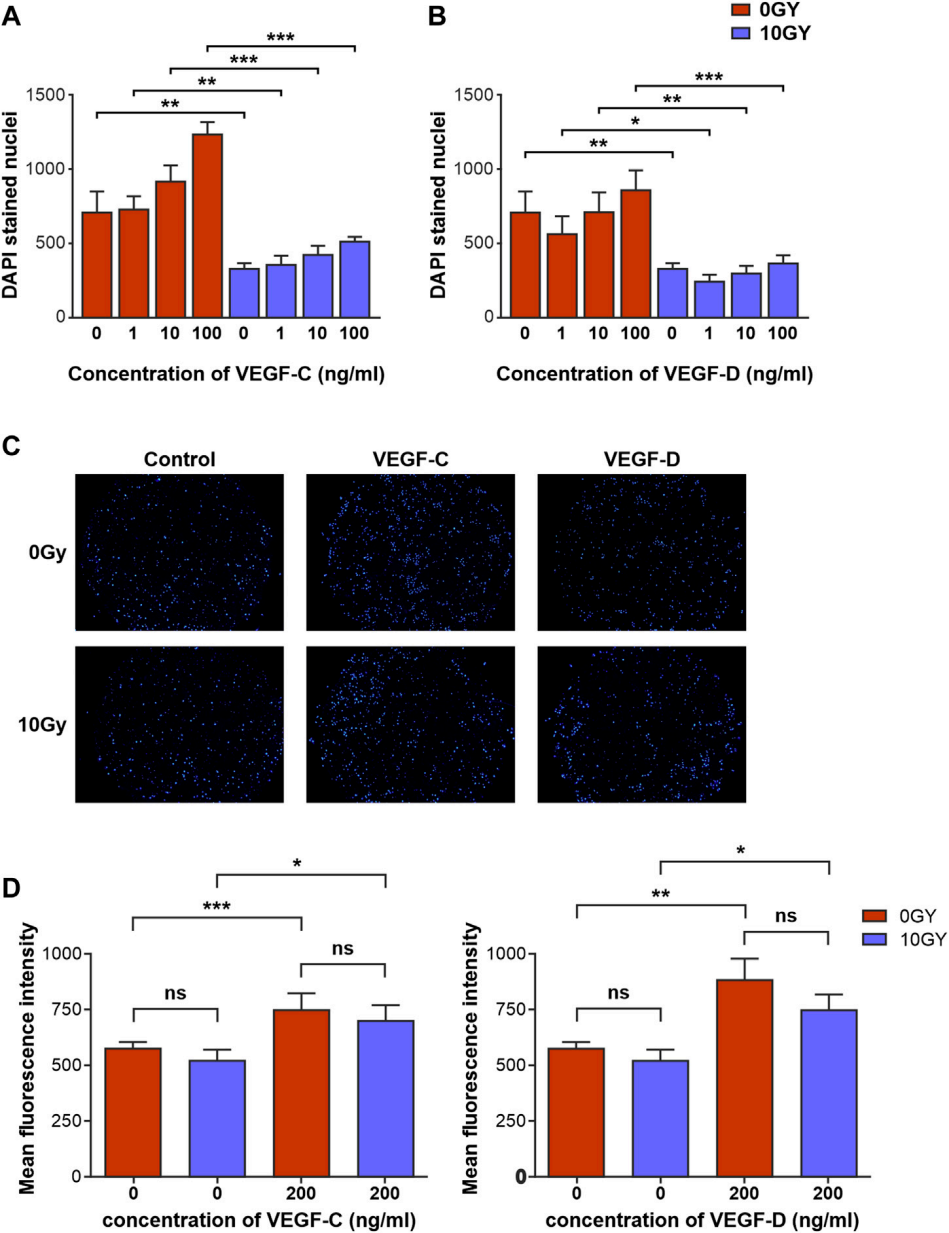
Radiotherapy differentially alters responsiveness of LEC to classical lymphangiogenic growth factors *in vitro*. Quantification of the effects of various concentrations of **(A)** VEGF-C and **(B)** VEGF-D on proliferation of 0 Gy and 10 Gy LEC. **(C)** Panel of DAPI stained 0 Gy and 10 Gy LEC representing transmigrated cells in response to control, VEGF-C and VEGF-D chemotactic gradients. **(D)** Quantification of 0 Gy and 10 Gy LEC chemotaxis in response to VEGF-C and VEGF-D. All assays performed a minimum of *n* = 3 times (with at least 3 technical replicates per assay). Data expressed as mean, with error bars representing SEM. *p* values calculated using one-way Anova. *p < 0.05; **p < 0.01; ***p < 0.001.

We next sought to investigate the mechanisms by which the reduction of lymphatic regeneration that we had observed in the above LEC functional assays may have occurred. We focussed on the lymphangiogenic factors VEGF-C and VEGF-D, which predominantly signal via VEGFR-3. Prior to growth factors being used in our cell-based assays, the activities of both VEGF-C and VEGF-D were confirmed using the established BAf assay (Stacker et al., 1999; Stacker et al., 2016) (Section 2.13). Both factors were shown to be potent in their ability to activate VEGFRs (Supplementary Figure S2). Firstly, we assessed the effect of VEGF-C and VEGF-D growth factors in our proliferation assays to establish a baseline of responsiveness. We found that treatment with VEGF-C and VEGF-D increased the proliferation rate of baseline unirradiated control (0 Gy) LEC (Table 1; Figures 3A, B) and demonstrated an increase in LEC proliferation with an increasing dose of growth factors (Figures 3A, B).

**TABLE 1:** The effect of VEGFC and VEGFD stimulation at 0 ng, 1 ng, 10 ng and 100 ng/mL concentrations, on the proliferation of 0 Gy and 10 Gy LEC. Data represented as mean ± SEM with assays completed with a minimum of 3 biological and technical replicates.

	0 Gy	10 Gy
VEGF C 0 ng/mL	702.80 ± 139.10	324.60 ± 34.75
VEGF C 1 ng/mL	722.80 ± 87.28	351.30 ± 56.42
VEGF C 10 ngml	911.90 ± 107.60	419.40 ± 56.34
VEGF C 100 ng/mL	1,229 ± 80.16	507.9 ± 29.46
VEGF D 0 ng/mL	702.8 ± 139.10	324.6 ± 34.75
VEGF D 1 ng/mL	560.8 ± 116.10	238.9 ± 45.62
VEGF D 10 ng/mL	707.1 ± 129.2	295.8 ± 46.89
VEGF D 100 ng/mL	854.20 ± 131.40	365.40 ± 49.01

In contrast, the potency of these growth factors at eliciting a proliferative response in irradiated 10 Gy LEC was significantly diminished (Table 1; Figures 3A, B). The response to VEGF-C and VEGF-D were diminished at all doses that were used to treat the cells. Interestingly, in keeping with our findings in earlier migration and chemotaxis assays being unaffected by radiotherapy (Figure 2E, G) chemotaxis assays performed with VEGF-C and VEGF-D showed that the responsiveness of LEC to these growth factors was not ameliorated by radiation (Figures 3C, D).

These findings demonstrate that radiotherapy impairs aspects of lymphangiogenesis in response to stimulation with VEGF-C and VEGF-D, the archetypal and most potent lymphangiogenic growth factors.

### 3.4 VEGFR-3 signalling is impaired in LEC after radiotherapy

**FIGURE 4:**
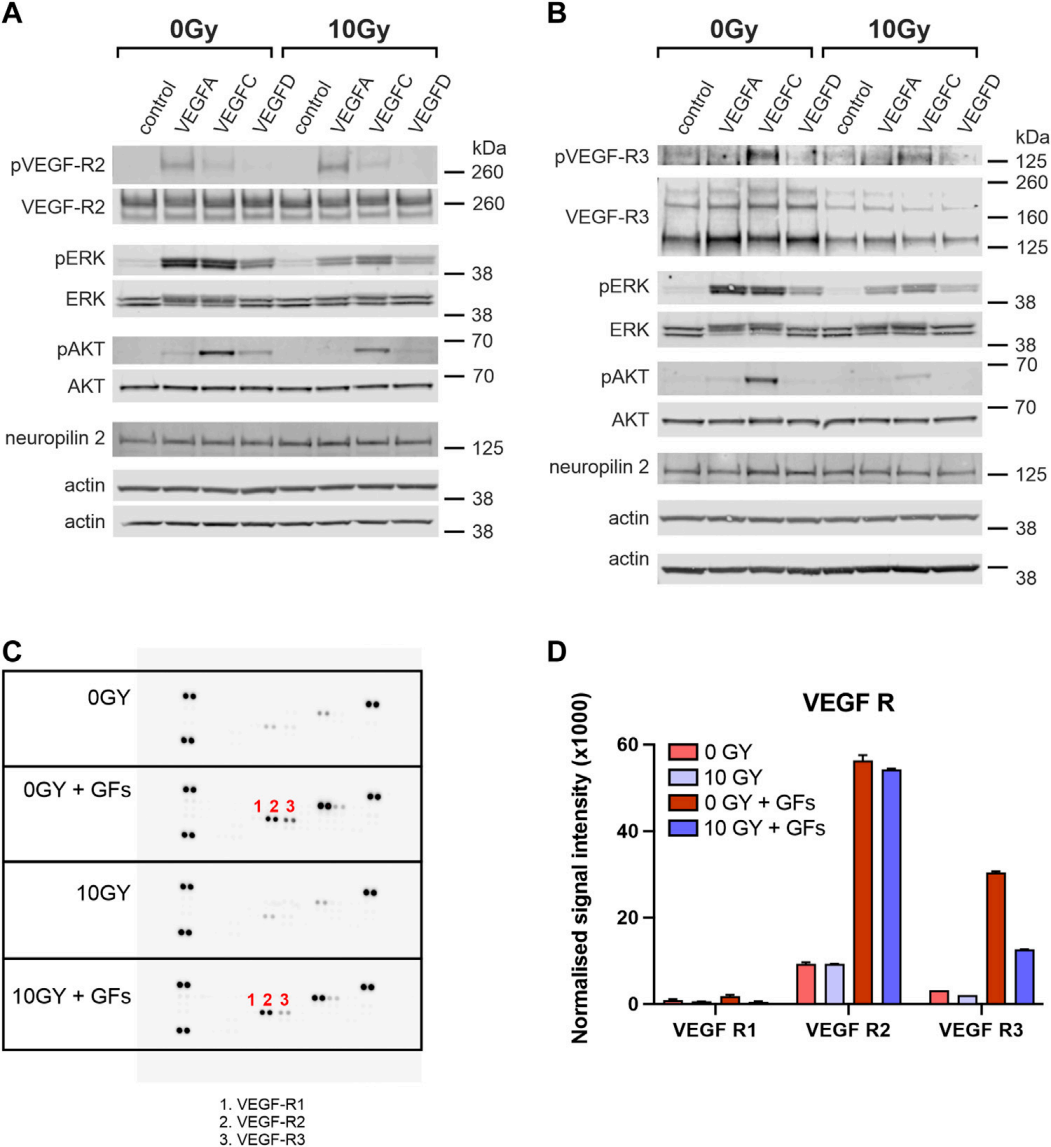
VEGFR-2 and VEGFR-3 activities are differentially altered with radiation treatment. Western blot analysis of cell lysates comparing levels of phosphorylated and total VEGFR-2 **(A)** and VEGFR-3 **(B)** in untreated (0 Gy) and irradiated (10 Gy) LEC stimulated with VEGF-A, VEGF-C or VEGF-D. Unstimulated cells served as a baseline control and VEGF-A is used as a control for VEGFR-2. The phosphorylation status of downstream signalling molecules ERK and AKT, as well as the levels of neuropilin2 (a VEGFR-3 co-receptor) and b-actin (loading control), are also shown. **(C)** Phospho-RTK antibody arrays were used to compare RTK activation in untreated and irradiated LEC. The identity of relevant phosphorylated RTKs is indicated by number: 1- VEGFR-1, 2 - VEGFR-2 and 3 - VEGFR-3. **(D)** The graph quantifies the normalized signal intensity and demonstrates an ameliorated VEGFR-3 signalling response in irradiated LEC compared to unirradiated controls, when stimulated with growth factors. Western Blot assays performed a minimum of 3 times, RTK assays were performed with 1 biological and 2 technical replicates. Data expressed as mean with error bars represented as SEM.

Tammela *et al.* previously demonstrated the nuanced relationship of the receptor tyrosine kinases (RTK)s VEGFR-2 and VEGFR-3 to vascular sprouting (Norrmén et al., 2011; Tammela et al., 2011). The ability of LEC to respond to VEGF-C or VEGF-D signalling pathways can be determined by the strength and capability of these growth factors to activate VEGFR-2 and VEGFR-3 (Joukov et al., 1996; Achen et al., 1998; Haiko et al., 2008). We identified a differential and selective influence on lymphangiogenesis *in vitro*, as well as restricted lymphangiogenesis in animal ear and tail wound models subjected to irradiation. We next analysed signalling driven by VEGF-C and VEGF-D in normal and irradiated LEC. To investigate whether VEGFR-2 and VEGFR-3 expression on LEC, and their respective functions, might be altered by radiotherapy, LEC were stimulated with control media, VEGF-A (as a control for VEGFR-2 stimulation), VEGF-C and VEGF-D, 48 h after radiotherapy or 0 Gy control treatment. Western blots were performed to monitor the levels of VEGFR-2 and VEGFR-3 signalling pathway activation, to compare the degree of phosphorylated (activated) VEGFR-2 (Figure 4A) or VEGFR-3 (Figure 4B) present in each of 0 Gy and 10 Gy LEC.

We verified that VEGF-A treatment (acting as a control) led to strong phosphorylation of VEGFR-2, whereas VEGF-C led to less marked but evident phosphorylation, and VEGF-D treatment resulted in minimal phosphorylation on Western blotting for VEGFR-2 in control 0 Gy LEC (Figure 4A). Irradiation of LEC (10 Gy) led to no detectable alteration to this profile of VEGFR-2 phosphorylation (Figure 4A). Notably, levels of total VEGFR-2 were similar in unirradiated and irradiated samples (Figure 4A). Activation of ERK and AKT appeared to be independent of stimulation via VEGFR-2 and irradiated (10 Gy) LEC demonstrated little relationship between irradiation, VEGFR-2 activation, or the profile of downstream mediators (Figure 4A).

In terms of VEGFR-3, both unirradiated (0 Gy) and irradiated (10 Gy) LEC groups showed no phosphorylation of VEGFR-3 in response to VEGF-A stimulation (a negative control in this experiment), whilst in contrast, VEGF-C resulted in strong activation (phosphorylation) of VEGFR-3 following treatment of (0 Gy) control LEC, which was reduced in irradiated (10 Gy) LEC (Figure 4B). Significantly, all irradiated LEC groups demonstrated diminished overall VEGFR-3 protein levels (Figure 4B). While treatment of LEC with 10 Gy radiation resulted in dampening of receptor activation by VEGF-C, VEGF-D resulted in minimal stimulation in comparison (Figure 4B). To determine if the dampened signalling observed was due to an overall reduction of VEGFR-3 levels, we quantified the ratio of pR3/tR3 from our Western blots (Table 2). We found that the ratio of tpR3/tR3 was constant, supporting the notion that reduced downstream signalling observed in our study was likely due to a total reduction of VEGFR-3 protein levels. To further validate this finding, qPCR was performed. Analysis post radiotherapy treatment (10 Gy) demonstrated a significant reduction in mRNA expression of VEGFR-3 (Supplementary Figure S3) when compared to the respective 0 Gy control groups. Together, these data indicate a specific radiation-induced dampening of the responsiveness of LECs to stimulation with VEGF-C via VEGFR-3, which is mediated by reduced levels of VEGFR-3.

**TABLE 2:** Ratios of pR3/tR3 from quantification of the optical densities of the signals from phosphorylated and total VEGFR-3 in Western blots. Western Blot assays performed a minimum *n* = 3.

Treatment	Ratio pR3/tR3—0 GY	Ratio pR3/tR3 10 GY
-ve control	0.604835	0.436316
+ VEGF-A	0.808624	0.94482
+ VEGF-C	1.191534	1.124148
+ VEGF-D	0.770186	0.827795

### 3.5 Downstream activation of ERK and AKT in response to growth factor mediated stimulation

Phosphorylation of both downstream ERK and AKT in response to VEGF-C stimulation were both reduced in following radiation treatment (Figure 4B). Critically, the expression of the VEGFR-3 co-receptor Neuropillin2 (Nrp2) was unaltered by radiation, suggesting that VEGFR-3 as a protein may be more susceptible to the effects of radiation injury.

To examine the possibility that the dampening effect of radiation on VEGFR-3 might extend broadly to other receptor tyrosine kinases, we analysed the activation of a variety of kinases, including Insulin R, FGFR, EGFR, PDGFR, RET, ROR, NGFR, Musk, by stimulating with a mixture of growth factors (EGF, bFGF, IGF-I, VEGF-A, VEGF-C and VEGF-D) added to LEC media using the RTK phosphoblot assay. We found that radiation did not elicit a universal response of dampening the activation of common RTKs with some receptors more sensitive or resistant to radiation than others. Interestingly, insulin R was the most sensitive to stimulation with the cocktail. (Figure 4C, D; Supplementary Figure S4). When we assessed VEGFR-2 in this assay, we showed that while it was stimulated in the unirradiated conditions, the response was not significantly diminished by radiation (Figure 4C, D, Supplementary Figure S4). In contrast, the introduction of the growth factor cocktail did stimulate VEGFR-3 in unirradiated conditions—however, VEGFR-3 stimulation was significantly dampened in the presence of irradiation, orthogonally demonstrating a reduction in the strength of VEGFR-3 signalling (Figure 4C, D, Supplementary Figure S4).

Together, these data indicate a specific radiation-induced dampening of the responsiveness of LEC to stimulation with key lymphangiogenic growth factors VEGF-C and VEGF-D via VEGFR-3. This effect is mediated by attenuated signalling of the tyrosine kinase receptor that corresponds to reduced levels of VEGFR-3 protein in irradiated LEC, in the setting of unaltered Nrp2 co-receptor expression levels.

### 3.6 Lymphangiogenesis in response to VEGF-C and VEGF-D stimulation is dampened by irradiation *in vivo*

Having determined that radiation selectively impairs VEGFR-3-mediated lymphangiogenesis *in vitro* (Figure 3), we sought to validate whether such a dampening of VEGFR-3 responsiveness to ligand stimulation would also hold true in our Prox-1/GFP mouse ear wounding models We have previously demonstrated that VEGF-C, secreted by VEGF-C over-producing tumors, was particularly able to elicit a dilatational response in unwounded ear lymphatics and a potent enhancement of lymphatic sprouting in mouse ear wound lymphatics, in the presence of both systemic and local VEGF-C (Shayan et al., 2007). Therefore, we treated control (0 Gy) and irradiated (10 Gy) mouse ears with VEGF-C and VEGF-D via local injection into a standardised area around the ear wounds.

**FIGURE 5:**
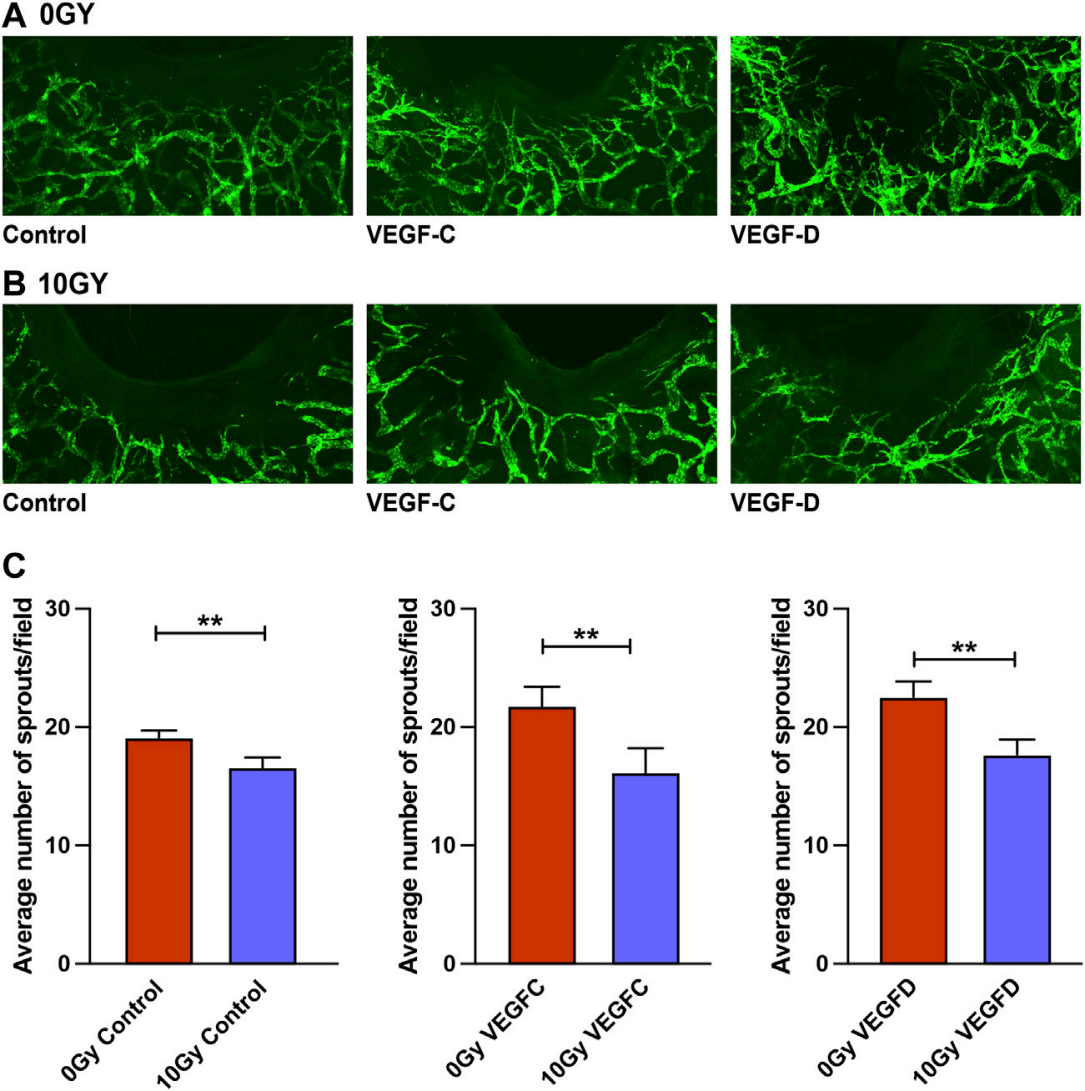
Treatment with VEGF-C and VEGF-D protein does not salvage lymphangiogenic impairment in Prox-1/GFP mouse ear wounds. **(A)** Prox-1/GFP mice ear wounding model in unirradiated ears with control (vehicle), 1 μg VEGF-C and 1 μg VEGF-D treatment showing a therapeutic lymphangiogenic response to these traditional growth factors. **(B)** Prox-1/GFP mouse ear wounding model in irradiated counterpart ears with control (vehicle), 1 μg VEGF-C and 1 μg VEGF-D treatment showing an ameliorated lymphangiogenic response to these traditional growth factors. **(C)** Quantification of lymphatic sprouting at the wound edge demonstrating differences between 0 Gy and 10 Gy wounded ears in response to vehicle, 1 μg VEGF-C and 1 μg VEGF-D treatments. Each group is represented by *n* = 6, with blinded quantification completed by two individuals independently. Data expressed as mean with error bars represented as SEM, *p < 0.05; ***p < 0.001.

The control unirradiated ear wound (Figure 5A) demonstrated lymphangiogenesis from pre-existing initial lymphatics that form a neo-lymphatic network that grew directionally toward the wound edge. When irradiated, this lymphangiogenic response was grossly ameliorated, with few neo-lymphatic sprouts seen in the area between the existing lymphatics and the wound edge (Figures 1B, C, 5B). We found that the lymphangiogenic response to VEGF-C and VEGF-D administration was diminished markedly in irradiated animals (Figures 5B, C), in comparison to the response to stimulation observed in their unirradiated counterparts. Quantification of the Prox-1/GFP mouse ear wounds demonstrated the lack of salvage of lymphangiogenesis when irradiated ears were treated using VEGF-C or VEGF-D.

Overall, this *in vivo* data builds on the earlier *in vitro* assays that showed selective inhibition of lymphangiogenesis along with selective attenuation of the VEGFR-3-mediated signalling pathway in radiation models; as well as diminished lymphatic responsiveness to lymphangiogenic factors VEGF-C and VEGF-D. These findings further support the hypothesis that reduced VEGFR-3 expression contributes to the deleterious effects of radiation treatment on the lymphatic vasculature.

## 5 Discussion

The lymphatic system facilitates immune responses, interstitial fluid homeostasis and clearance of waste products (Oliver et al., 2020). Radiotherapy used in the oncological management of human tumours can lead to impairment in the lymphatic system that results in infections, poor healing, and secondary lymphoedema (Ji, 2008). The combination of surgery and radiotherapy has proven deleterious to the ability of lymphatic vessels to recover and heal, with a rate of lymphoedema of up to 60%. However, to date, the mechanisms behind the resulting secondary lymphoedema have remained elusive. Therefore, we used a combination of *in vitro* and *in vivo* models to investigate how radiotherapy specifically neuters the ability of lymphatics to recover from injury. We further studied VEGFR-2 and VEGFR-3 responsiveness to lymphangiogenic growth factors in the aftermath of radiotherapy and examined the ability of these growth factors to rescue the injury to LEC and lymphatic vessels *in vitro* and *in vivo*. Finally, we investigated the molecular mechanisms underpinning the impairment of LEC functions and lymphangiogenesis seen in our models.

We found that radiotherapy impaired lymphangiogenesis in mouse ear and tail wounding models. Not only did radiotherapy significantly abrogate early lymphatic regeneration in these wounding models, but the potentiating effect of combined therapy was manifested as greater volume increases in the tail lymphoedema model. Whilst previous literature had confirmed that radiotherapy has mixed effects on quiescent lymphatics (Avraham et al., 2010; Jackowski et al., 2010; Kesler et al., 2013), the effect of radiotherapy on lymphangiogenesis in wounds shown in our study is an important novel finding. Our data also resonates with clinical studies in which the incidence and severity of lymphoedema is increased with combined surgery and radiotherapy (Johnson et al., 2019); and the specific diminution of lymphatic vessel sprouting supports the importance of lymphatic vessel regenerative capacity for the ability to overcome iatrogenic injury to the lymphatic system, as is incurred during this treatment modality.

The formation of lymphatic vessels requires orchestrated mechanisms that when activated induce opposing proliferative and migratory cellular responses of LECs, as well as inducing sprouting and cell migration at the same time as they block proliferation and *vice versa*, illustrating the fine balance critical for the formation of functional vessels (Mühleder et al., 2021). To understand why radiation-induced impairment of lymphangiogenesis occurs, we investigated the effects of a single 10 Gy dose of radiotherapy on LEC functions *in vitro*. Traditional theories of radiotherapy soft tissue injury postulated a lethal and obliterative effect on normal soft tissues and cells that lay in the path of external radiation beams that target cancer (Dormand et al., 2005). While Day et al. (2014) had previously found that radiotherapy altered blood vessel endothelial replication, Narayanan et al. (2018) identified altered barrier function in irradiated LEC. Surprisingly, we detected a selective impairment of key LEC cellular functions, rather than a universal functional impairment or obliterative response. Namely, migration and chemotaxis functions remained unimpaired whilst key lymphangiogenic processes such as LEC sprouting, tube formation and proliferation were selectively reduced after radiation treatment, in *in vitro* assays. It is plausible, therefore, that the effect radiotherapy has on LEC proliferation impacts the induction of sprouts given that stalk cells guided by tip cells undergo proliferation as part of the sprouting process (Mühleder et al., 2021). Changes to these dynamic cellular functions correlated with the histological analysis undertaken by Avraham et al. (2009) on irradiated murine tissue, which demonstrated ectatic and phenotypically abnormal lymphatic vasculature. This loss of coordination of important lymphangiogenic processes is likely to contribute to failure of formation of a working lumen and maintenance of vessel patency that contribute to vital processes in fluid homeostasis (Allam et al., 2020), especially when challenged by iatrogenic or accidental wounding in the irradiated region. It is noteworthy that other cell types within the soft tissue, such as fibroblasts, have been shown to have both positive and negative effects on lymphangiogenesis (Wang et al., 2020). We have previously demonstrated that radiotherapy affects fibroblast functionalities and other cells that compromise the soft tissue, and this may either directly or indirectly influence the promotion of lymphangiogenesis (Shukla et al., 2020; Shukla et al., 2022). It is therefore possible that the *in vivo* effects observed in this study could, in part, be attributed to such influences.

Building on these preliminary findings of selective impairment of lymphangiogenic functions that lead, in turn, to impaired lymphangiogenesis *in vivo*, we sought to further study the cellular mechanism underpinning the sublethal radiation injury to LEC. The most well-studied lymphangiogenic growth factors are VEGF-C and VEGF-D and their receptor VEGFR-3 (Partanen et al., 1999; Stacker et al., 2014). It has been postulated that delivery of VEGF-C and VEGF-D may be able to promote regeneration of LEC via activation of the VEGFR-2 and/or VEGFR-3 RTKs, and thus may be a useful approach by which to treat lymphoedema (Szuba et al., 2002; Cheung et al., 2006; Ji, 2007). VEGF-C delivery by several methodologies, including recombinant protein, viral vector, naked plasmid, or topical therapy transiently improved surgically induced lymphoedema in several animal species, using various lymphoedema models (Stacker et al., 2014; Brown et al., 2022). Goldman et al. (2005) demonstrated that VEGF-C over-expression in a mouse tail skin regeneration model, resulted in lymphatic hyperplasia that subsided after withdrawal of VEGF-C stimulation (Oliver et al., 2020). Jackowski et al. (2010) reported increased expression of CD68^+^/VEGF-C^+^ macrophages in irradiated skin 2–8 weeks after radiotherapy. This influx, thought to represent an endogenous attempt at inducing lymphangiogenesis after lymphatic injury, did not circumvent the development of chronic lymphedema. Moreover, an *in vitro* study by Kesler et al. (2013) demonstrated that VEGF-C treatment of LEC prior to radiotherapy was not radioprotective and resulted in reduced levels of lymphangiogenesis. This observation may, in part, be explained by our findings. We demonstrated reduced levels of VEGFR-3 in response to radiation, which in turn led to reduced levels of phospho-VEGFR-3 in response to treatment with VEGF-C and subsequent dampening of downstream signalling cascades. It would be of interest to test the effect of blocking VEGFR-3 signalling by treatment with a neutralising VEGFR-3 antibody to understand the extent of VEGFR-3 signalling within the microenvironment following radiation injury. Meanwhile, neither the expression or phosphorylation of VEGFR-2 was altered in response to radiation, nor was the expression of the VEGFR-3 co-receptor Nrp2. The alterations to VEGFR-3 protein demonstrated *in vitro* are from an early timepoint of 48 h, and attenuated response to VEGF-C and VEGF-D treatment *in vivo* was evident across the 3-week experimental period despite no ongoing radiation treatment. This suggests the irradiation mediated alteration of the VEGFR-3 signalling axis may represent more than a transient change and may impact long-term lymphatic repair. Interestingly, Harris et al. (2022) demonstrated that VE-Cadherin is necessary for VEGFR-3 surface expression within cardiac lymphatics, which influences LEC responsiveness to VEGF-C stimulation. It could be important to evaluate any possible effects on VEGFR-3 surface expression in the setting of radiotherapy. Thus, a detailed mechanistic study would be required to fully understand the reduced VEGFR-3 signalling in the current study.

The application of lymphangiogenic growth factors that act via VEGFR-3 to improve lymphatic function after radiation is unlikely to result in normal levels of regenerative/reparative lymphangiogenesis due to both a selective amelioration of lymphangiogenesis and lymphatic response to VEGF-C and VEGF-D. This finding may help to explain the low success of adapting the most potent pro-lymphangiogenic growth factor, VEGF-C, to develop therapeutics to stimulate the lymphatic system in the setting of radiotherapy injury, secondary lymphoedema or enhance wound healing; noting also the added complexity that VEGF-C treatment can lead to heterodimerisation between VEGFR-2 and VEGFR-3, a phenomenon that does not occur with VEGF-A treatment, (Secker and Harvey, 2021). It is tempting to speculate that the reduced VEGFR-3 signalling upon VEGF-C stimulation following radiotherapy may be attributed to a potential alteration in the ratios of homo-to-heterodimer formation of VEGFR-3, although this hypothesis warrants further mechanistic exploration.

Our data may help inform the field regarding so-far disappointing application of VEGF-C-based therapy in the setting of lymphoedema. Whilst a 24-month Phase I trial of 15 patients treated with adenoviral-mediated VEGF-C (Lymfactin^®^) delivery in combination with free lymph node transfer for the treatment of secondary lymphoedema of the upper limb suggested promising results (Leppäpuska et al., 2022), results are pending from a 39-participant double blinded, placebo-controlled randomised phase II trial of (Lymfactin^®^) to demonstrate an objective clinical improvement in lymphoedema of the upper limb (Clinical study with Lymfactin^®^ in the treatment of patients with secondary lymphedema—trial reference *NCT0365896*7). Our study may help inform the choice of suitable patients for any future clinical trials by this treatment modality.

Previous studies noted the paradoxical finding of increased VEGF-C expression in lymphedematous tissue (which may lead to increased leaky neo-vasculature), via mechanisms that remain largely unknown; (Jensen et al., 2015), that may indicate a positive-feedback response to compensate for diminished VEGFR-3 activation. VEGFR-3 activation by VEGF-C and/or VEGF-D signalling is vital to the processes of LEC sprouting, proliferation and migration, and VEGFR-3 mutations (Milroy Disease) lead to congenital and infantile lymphedema (Secker and Harvey, 2021). Mäkinen et al. (2001) described transgenic mice expressing inactive VEGFR-3 also demonstrated hindlimb lymphedema-like swelling and fibrosis, characterised by regressed lymphatic vessels and impaired lymphangiogenesis. The findings of our study suggest that radiotherapy also leads to an “acquired” attenuation of VEGFR-3 signalling and may serve to provide a better understanding of radiation-induced lymphoedema, in terms of previous clinical and experimental observations of VEGFR-3 dysregulation. We demonstrated, for the first time, molecular mechanisms driving radiotherapy-induced lymphatic dysfunction and why stimulation with traditional lymphangiogenic factors may be unable to initiate reparative processes and mediate signalling through an impaired VEGFR-3 axis.

## Conclusion

Our novel findings shed light on the mechanisms behind radiotherapy induced lymphatic injury. Challenging traditional dogma regarding a purely obliterative/lethal nature of radiotherapy injury (Lenzi and Bassani, 1963; Ariel et al., 1967), we demonstrated that radiotherapy does not universally ablate lymphatics, but selectively impairs lymphangiogenesis in the setting of healing lymphatics in irradiated tissue. We show that the molecular mechanisms behind these changes include a selective obliteration of LEC responsiveness to VEGF-C and VEGF-D via the VEGFR-3 signalling axis in response to radiotherapy exposure, due to downregulation of VEGFR-3 protein levels, with a consequentially decreased VEGFR-3 phosphorylation and downstream signalling cascades within irradiated LEC. Furthermore, we provide evidence of why potent VEGF-C-based therapy alone is unlikely to be therapeutically beneficial in reversing the radiation-induced injury to LEC and the lymphatics that they constitute, and therefore, also in treating the resulting condition of lymphoedema in patients that have undergone surgery and radiation therapy to treat their cancer. It is hoped that these data will enhance our understanding of mechanisms of radiation-induced lymphatic vessel injury and lead to exploration of alternative and improved therapeutic approaches in the treatment of the secondary lymphoedema and the devastating impact of this disease on the lives of cancer survivors.

## Data Availability

The original contributions presented in the study are included in the article/supplementary material, further inquiries can be directed to the corresponding author.

## Abbreviations

EC, endothelial cells; LEC, lymphatic endothelial cell; RTX, radiotherapy; VEGF, vascular endothelial growth factor.
