# Supplementary figures and images for: Radiation therapy attenuates lymphatic vessel repair by reducing VEGFR-3 signalling
Source: Front Pharmacol. 2023 Apr 28;14:1152314. doi: 10.3389/fphar.2023.1152314 (PMC10176020; doi:10.3389/fphar.2023.1152314)

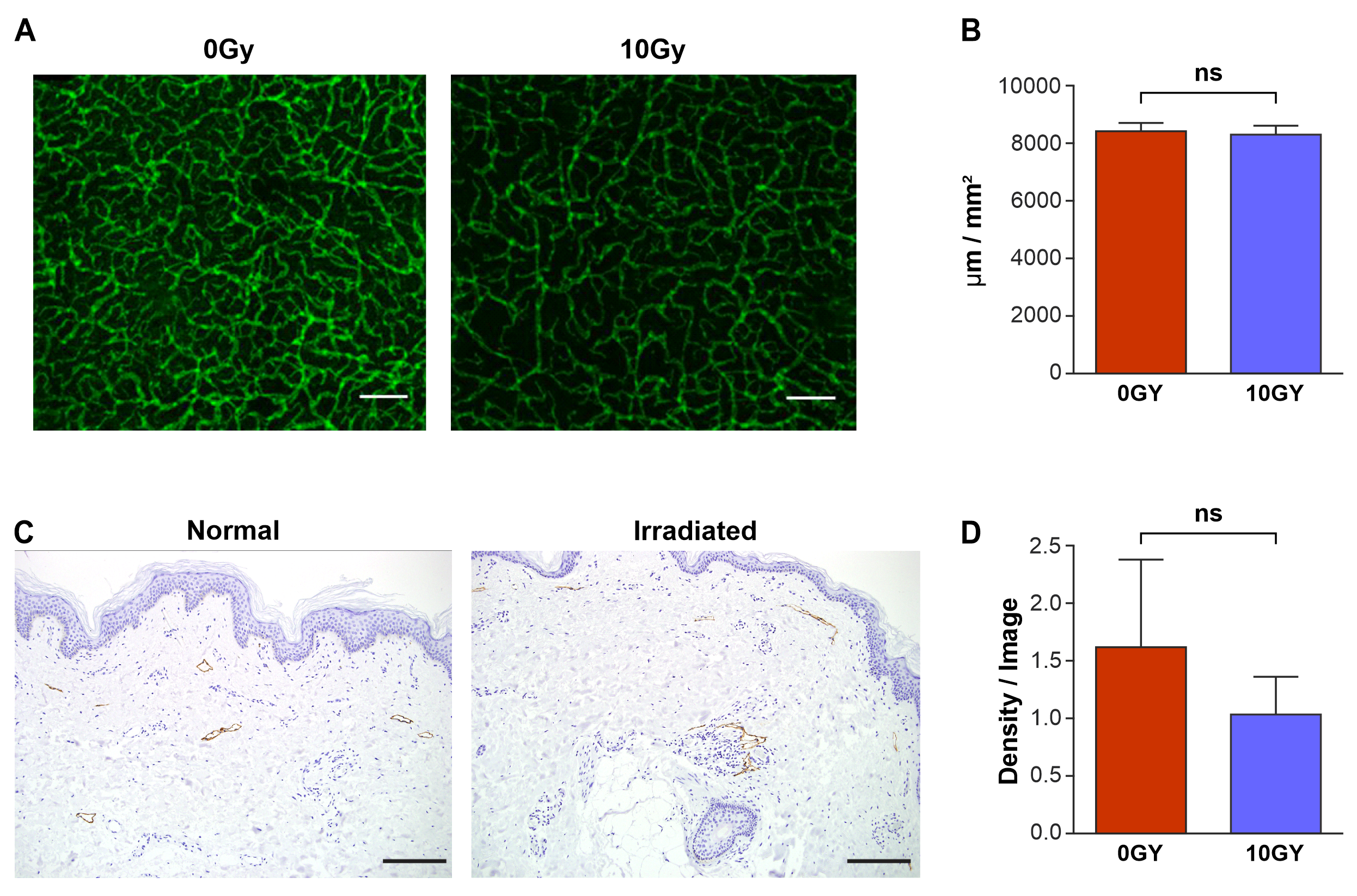

Supplement: Supplementary file 1 [file Presentation1.zip › Figure S1.tif]

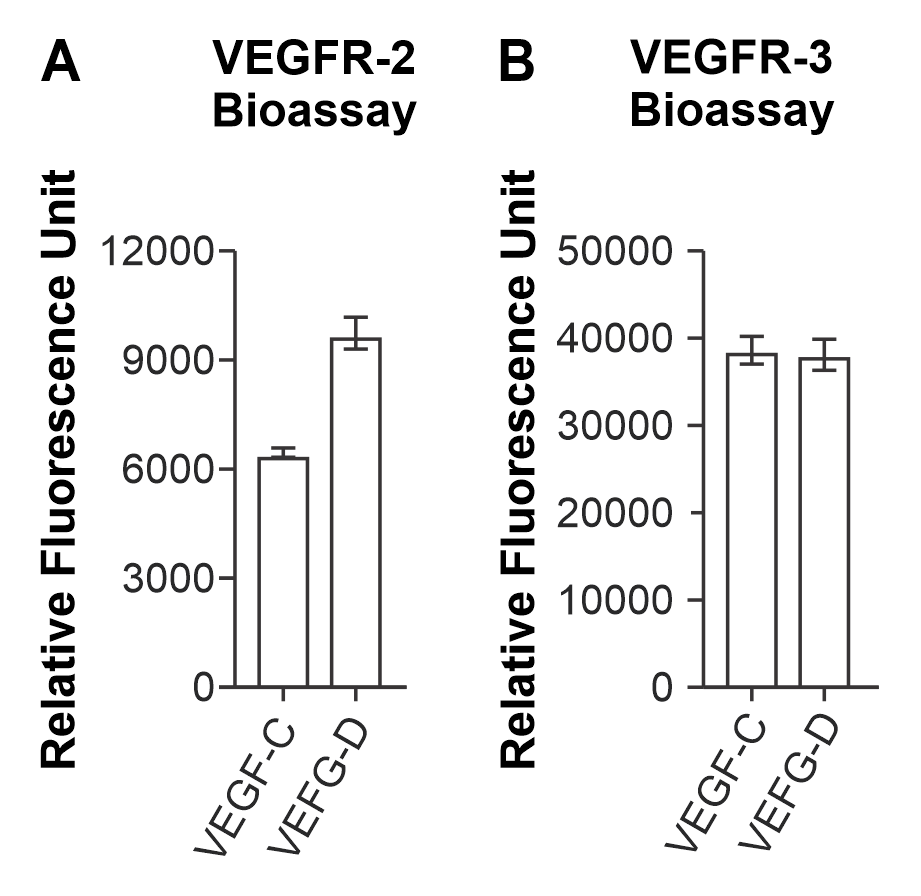

Supplement: Supplementary file 1 [file Presentation1.zip › Figure S2.tif]

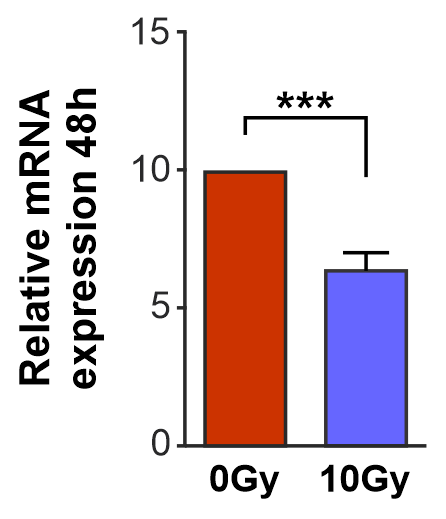

Supplement: Supplementary file 1 [file Presentation1.zip › Figure S3.tif]

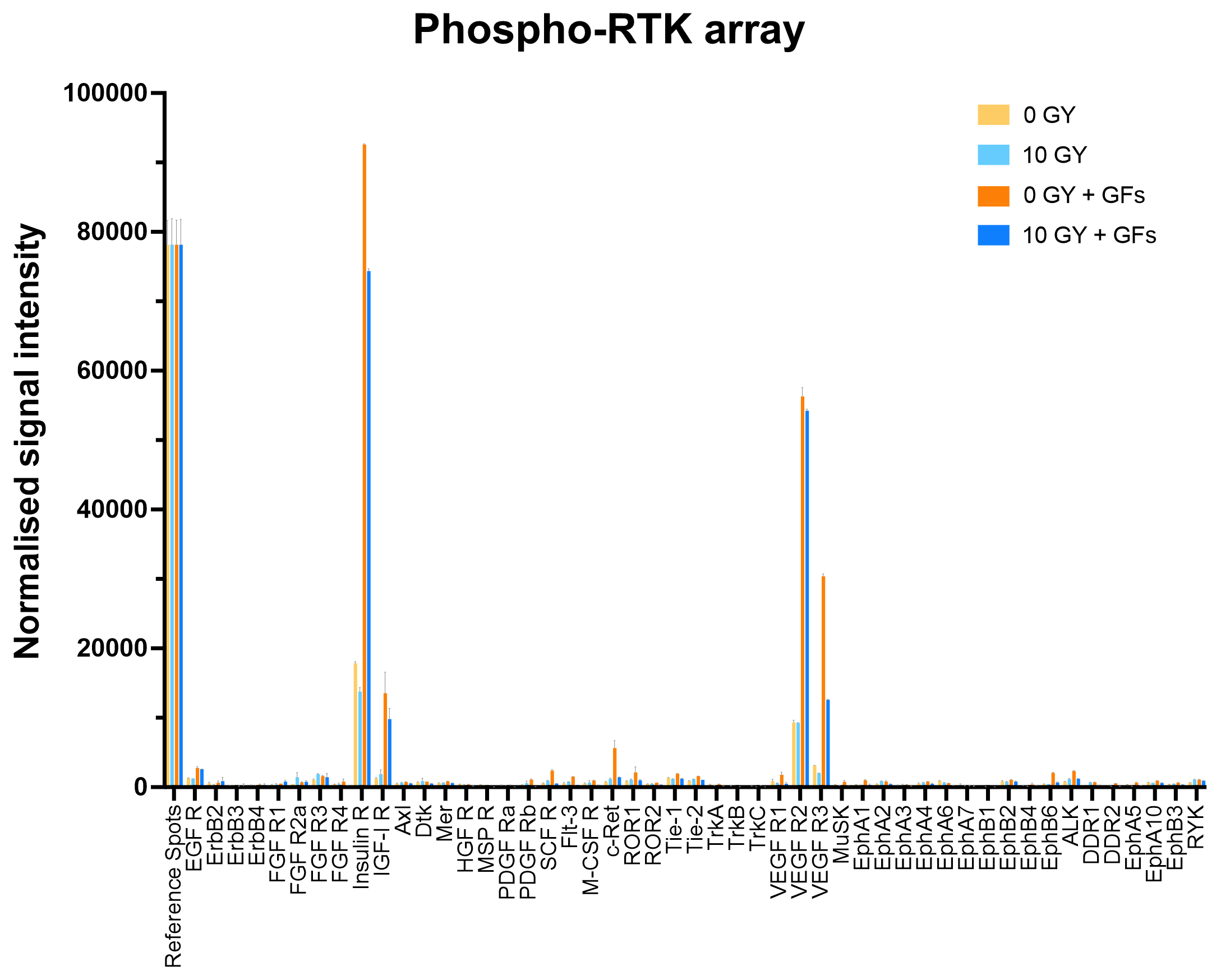

Supplement: Supplementary file 1 [file Presentation1.zip › Figure S4.tif]
